# A shared behavioral framework for assessing preserved cognitive function in aging in rodent models

**DOI:** 10.1002/alz.087235

**Published:** 2025-01-09

**Authors:** Audrey E Branch, Joseph McQuail, Catherine C. Kaczorowski, Thomas C Foster, John F. Disterhoft, Matthew J. Huentelman, Peter R. Rapp, Carol A Barnes

**Affiliations:** ^1^ Johns Hopkins University, Baltimore, MD USA; ^2^ University of South Carolina, Columbia, SC USA; ^3^ Tufts University School of Medicine, Boston, MA USA; ^4^ The Jackson Laboratory, Bar Harbor, ME USA; ^5^ University of Michigan, Ann Arbor, MI USA; ^6^ University of Florida, Gainesville, FL USA; ^7^ Northwestern University Medical School, Chicago, IL USA; ^8^ Neurogenomics Division, Translational Genomics Research Institute, Phoenix, AZ USA; ^9^ The Translational Genomics Research Institute (TGen‐ an Affiliate of City of Hope), Phoenix, AZ USA; ^10^ National Institute on Aging/National Institutes of Health (NIA/NIH), Baltimore, MD USA; ^11^ University of Arizona, Tucson, AZ USA

## Abstract

**Background:**

Animal models provide a valuable basis for identification of conserved pathological substrates and processes underlying age‐related diseases as well as neurobiological features supporting cognitive resilience in the aging brain. Behavioral measures are a fundamental component in the assessment of cognitive processes but are rarely standardized across laboratories. Currently, there is a scarcity of centralized and standardized data infrastructure for behavioral experiment data collected across laboratories which presents a barrier for data sharing, hypothesis generation, and collaboration. A key goal of the Reserve and Resilience Collaboratory has been to create consensus approaches and definitions for the study of cognitive aging. We have designed a standard reporting framework and a centralized database across several labs.

**Method:**

In rodent model studies of cognitive aging trajectories, the Morris water maze spatial learning paradigm is a widely used test for characterization of age‐ and disease‐related cognitive decline. The place learning version of this test depends on intact limbic and cortical systems which are key sites of dysfunction in both healthy aging and late life neurodegenerative diseases. Importantly, learning index and memory scores derived from Morris water maze testing in aged subjects replicate individual differences observed in human subjects and provide a basis for identifying covarying neurobiological changes supporting cognitive reserve and maintenance. Given the prevalence of this test for characterization of individual cognitive aging trajectories and preclinical intervention studies we have designed a standard reporting framework and centralized database housing Morris water maze behavioral data collected from rodent models across several labs.

**Result:**

Four laboratories have committed behavioral measures to the database allowing meaningful comparisons to be made across rodent strains. Initial comparisons will include variables relating to sex differences, strain, and age of testing.

**Conclusion:**

As we develop this resource, we intend to include access to biological measures, tissue banks, and additional behavioral measures taken from subjects in the database. Our goal is to provide a resource to enable collaborations and initiatives to advance the study of cognitive and brain reserve in aging, toward the prevention of Alzheimer’s disease.

